# A Review on SERS-Based Detection of Human Virus Infections: Influenza and Coronavirus

**DOI:** 10.3390/bios11030066

**Published:** 2021-02-28

**Authors:** Fernanda Saviñon-Flores, Erika Méndez, Mónica López-Castaños, Alejandro Carabarin-Lima, Karen A. López-Castaños, Miguel A. González-Fuentes, Alia Méndez-Albores

**Affiliations:** 1 Facultad de Ciencias Químicas, Benemérita Universidad Autónoma de Puebla, 72570 Puebla, Mexico; fernanda.savinonfl@alumno.buap.mx (F.S.-F.); erika.mendez@correo.buap.mx (E.M.); miguel.gonzalezfuentes@correo.buap.mx (M.A.G.-F.); 2 Facultad de Ciencias Biológicas, Benemérita Universidad Autónoma de Puebla, 72570 Puebla, Mexico; monica.lopezca@alumno.buap.mx; 3 Centro de Investigaciones en Ciencias Microbiológicas, Benemérita Universidad Autónoma de Puebla, 72570 Puebla, Mexico; alejandro.carabarin@correo.buap.mx; 4 Centro de Química-ICUAP-Posgrado en Ciencias Ambientales, Benemérita Universidad Autónoma de Puebla, 72570 Puebla, Mexico; karen.lopezc@alumno.buap.mx

**Keywords:** SERS, quantification strategies, anthropozoonosis, virus, zoonosis, influenza, coronavirus, COVID-19

## Abstract

The diagnosis of respiratory viruses of zoonotic origin (RVsZO) such as influenza and coronaviruses in humans is crucial, because their spread and pandemic threat are the highest. Surface–enhanced Raman spectroscopy (SERS) is an analytical technique with promising impact for the point–of–care diagnosis of viruses. It has been applied to a variety of influenza A virus subtypes, such as the H1N1 and the novel coronavirus SARS−CoV−2. In this work, a review of the strategies used for the detection of RVsZO by SERS is presented. In addition, relevant information about the SERS technique, anthropozoonosis, and RVsZO is provided for a better understanding of the theme. The direct identification is based on trapping the viruses within the interstices of plasmonic nanoparticles and recording the SERS signal from gene fragments or membrane proteins. Quantitative mono- and multiplexed assays have been achieved following an indirect format through a SERS-based sandwich immunoassay. Based on this review, the development of multiplex assays that incorporate the detection of RVsZO together with their specific biomarkers and/or secondary disease biomarkers resulting from the infection progress would be desirable. These configurations could be used as a double confirmation or to evaluate the health condition of the patient.

## 1. Introduction

Infectious diseases such as influenza (flu) and coronaviruses (CoV)—including the 2019 coronavirus disease (COVID-19)—infect millions worldwide, causing hundreds of thousands of deaths [1]. These viruses are transmitted by person-to-person contact when someone touches a surface that is contaminated with respiratory germs and then touches their mouth or nose and by the inhalation of small droplets exhaled by infected persons as a result of coughs or sneezes [2]. Clinical characteristics of patients with the flu share similarities with those of symptomatic patients infected by COVID-19 and include the presence of dyspnea, cough, and fever, resulting in medical complications such as pneumonia and kidney injury. Therefore, a proper diagnosis through sensitive and accurate analytical techniques or rapid tests is mandatory for the adequate management of patients [3]. Nowadays, there is an urgent need to develop and consolidate efficient analytical methods capable of the reliable detection and identification of viruses that cause human infections. This necessity is especially significant in the case of viruses of animal origin (zoonoses) with a potential pandemic threat, such as influenza and coronaviruses.

Up to now, real-time quantitative polymerase chain reaction (PCR) has been the gold standard lab method for virus identification/quantification [4]. The basis of this technique is the amplification of a region of DNA from a minimal concentration of DNA template that, in the case of viruses, would consist of certain regions of their genome. The analyses of the amplified products is carried out in real time, and this is done by using fluorescent primers or DNA binding fluorophores working as the reporter component [5,6].

Real-time PCR presents high sensitivity, specificity, robustness, experimental reliability, and the possibility to identify multiple members of a particular family, strain, or pathotype. However, in addition to the complex setup of the technique, it requires long sample preparation times (purification and extraction of DNA or RNA). On the other hand, multiple DNA target molecules are necessary to allow a correct amplification in the PCR reaction, and in many cases, several tissue samples are required from the same patient; otherwise, the result can be erroneous if the correct sample is not taken [5,7].

Although mobile-based diagnoses (point-of-care testing systems, POCT) are faster and easier to use than PCR, they can present less sensitivity, accuracy, and reliability; for this reason, PCR presents a superior performance [8]. In a pandemic, these drawbacks can be a big problem for the prevention and control of outbreaks. Additionally, a late diagnosis and unnecessary treatments by a misdiagnosis results, in some cases, in a reduction of life expectancy for the infected individuals.

Since its discovery in 1973, surface-enhanced Raman spectroscopy (SERS) has advanced dramatically and gained a position as one of the great promises to be used as a reliable POCT for various analytes, including viruses [9,10]. With SERS, it is possible to detect analytes at ultra-low concentrations and carry out measurements without sample preparation. If the technique is combined with an immunoassay strategy, a high specificity is achieved [11]. Additionally, different analytes can be screened by multiplex SERS immunoassays, which undoubtedly favor an increase in the versatility of the technique, unlike PCR, which only allows testing samples with genetic material [12,13].

In this review, an analysis of the SERS strategies for addressing the identification/quantification of infectious diseases of zoonotic origin, influenza (flu) and coronaviruses (CoV), including COVID-19, is presented. In addition, we provide a brief introduction to the SERS technique and a description of zoonosis and the factors that promote them, as well as a medical and historical background of the respiratory viruses of zoonotic origin (RVsZO).

## 2. Principles of SERS

### 2.1. Enhancement Mechanisms

SERS is based on the amplification of the Raman response of an analyte interacting with the surface plasmon of metals such as Au, Ag, or Cu; in some cases, the response result is enough to achieve a single-molecule detection [14]. Paradoxically, after 46 years since the discovery of SERS, the exact mechanism in the signal enhancement is still a matter of debate, but it is generally accepted to be driven mainly by two principles: the electromagnetic (EM) and the chemical (CM) effects. These mechanisms have already been deeply explained in the literature [15,16,17].

**Figure 1:**
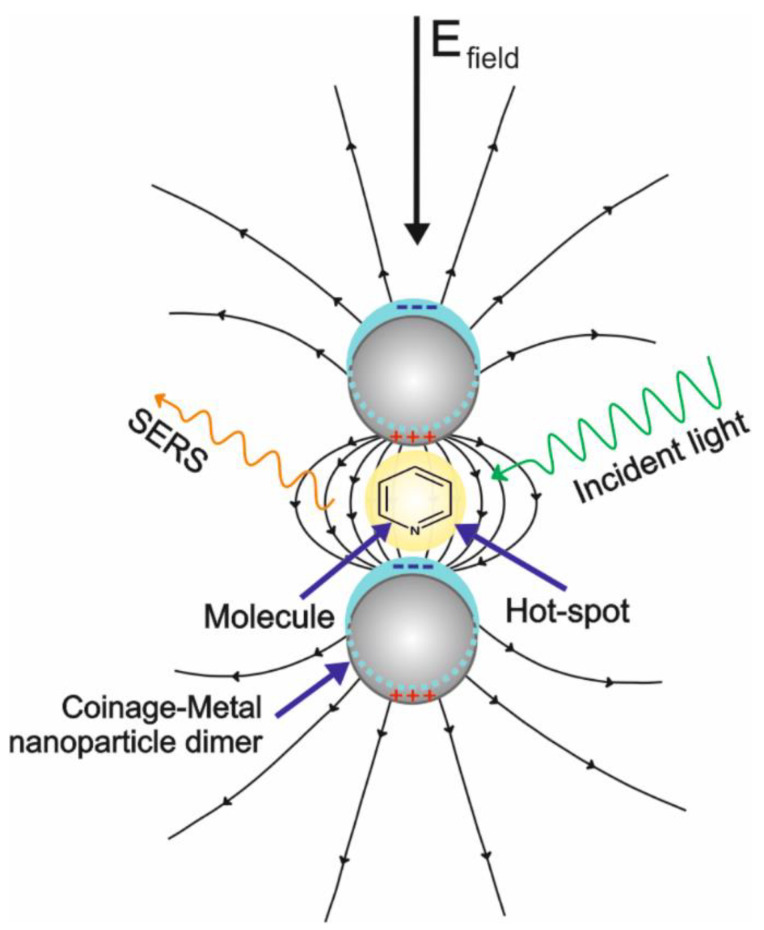
Schematic representation of the surface-enhanced Raman scattering (SERS) from the electromagnetic (EM) effect.

In general, the EM mechanism is the most understood; it comes from the substrate and is originated when a free electron-like metal is irradiated with a laser whose frequency is resonant with the frequency resulting from the collective oscillation of conduction band electrons. This phenomenon is known as surface plasmon resonance (SPR) [15]. Especially in some regions called hot spots, an intense local field enhancement is produced around the metal interface by the concentration of light, which creates an oscillating dipole on the molecules in close proximity with the nanoparticles; this results in an oscillating dipole that enhances the radiation efficiency (Figure 1) [15,16]. In this situation, the magnitude of the electromagnetic field between the nanoparticle center and the analyte decays with distance. It is considered that the maximum enhancement is obtained in a distance that is not greater than 2 nm [18]. Therefore, to obtain the maximum enhancement by this principle (about eight orders of magnitude), the physical interaction between the analyte and the substrate or the specific adsorption of a small-sized analyte is required [19].

The chemical mechanism (CM) involves the intrinsic features of the adsorbate and the new properties arising when the adsorbate is combined with the substrate (adsorbate–metal nanostructure complex) under the effect of the incident light that results in a modification of the molecular polarizability [18]. The plasmonic nature, substrate roughness, or nanoparticulate structure is not mandatory for the CM to occur, as, instead, the experimental conditions and the adsorbate orientation and symmetry on the surface have an influence [20]. Thus, the CM mechanism comes from different origins, some of those scarcely understood, and it is limited to occurring within the first layer of absorbed molecules (short-range interactions) [21]. So far, the main known contributions of the CM mechanism result from the resonant effect of the charge transfer (CT) between the metal and the target molecule or vice versa, with a Raman enhancement of about four orders of magnitude [22].

### 2.2. SERS Substrates

SERS substrates can be made of rough surface metals, as well as nanoparticles in suspension or deposited on solid substrates [23]. The use of colloids as SERS substrates is widely spread because of their ease of synthesis with good size and morphology control, in addition to the possibility of studying absorption phenomena in aqueous environments [24]. However, potential applications are restricted by their poor stability and reproducibility due to the Brownian motion of particles in the suspension that causes a permanent change in the measurement scenario. Immobilization of the nanoparticles on a solid substrate, usually silicon, provides a significant increase in the reproducibility of the enhancement effect; however, the current research in this area consists of the reproduction of the same substrate surface to increase the repeatability of the spectroscopic measurements [25]. Among the most efficient techniques to obtain organized metallic nanostructure arrays on solid substrates for SERS, we can find lithography and its indirect techniques (electron beam lithography, laser interference lithography, UV photolithography, and electro-oxidative lithography) as well as block copolymer self-assembly [26].

### 2.3. SERS Measurements on Solid Substrates

SERS measurements on solid substrates can be made in dry or wet forms [20]. The dry form is more common and is carried out after evaporation of the sample on the substrate. The evaporation process can be preceded by the immersion of the substrate into the sample for a certain period (incubation process) [27]. Wet experiments can be made either by using sophisticated microfluidic lab−on−a−chip SERS systems (LOC–SERS) or on a drop of the liquid sample placed on the SERS substrate that has been covered with a thin coverslip. It can also be carried out using the “drop technique”, which consists of the signal acquisition on the periphery of a drop of the sample placed on the SERS substrate [28]. Nevertheless, these methodologies present drawbacks that influence the final SERS results. In the evaporative procedure, the adsorption process can be abruptly interrupted during the sample evaporation, inducing heterogeneity in the formed layer or the “coffee ring” effect [29]. In addition, oxidation or dissolution of the metallic nanoparticles may occur during the incubation processes, together with the loss of molecular perpendicular arrangements during the Raman measurements. On liquid media, the use of another interface or the influence of different refractive indexes when using a coverslip gives a weak Raman signal. Concerning the “drop technique”, it has the advantage that a proper change in the substrate hydrophobicity can lead to the analyte preconcentration, thus avoiding the diffusion limitation and enhancing the SERS signal [30,31]. However, a camera in the Raman spectrometer is necessary to identify the optimal distance from the periphery to the center of the drop where the measurement must be recorded. This distance must be sufficient to avoid the effects from evaporation of the sample at the chosen spot during the experiment but short enough to obtain a good response from the substrates.

### 2.4. Detection of Analytes by SERS

The detection of analytes by SERS can be conducted by direct or indirect approaches [15]. The direct measurement is accomplished with the analyte adsorbed on the substrate or when the analyte is retained close enough to the substrate using molecular linkers or capture elements such as antibodies, aptamers, or related molecules immobilized onto nanostructured surfaces. This method is recommended for analytes that present a high Raman scattering cross-section. It has the advantages of the major control and accuracy of the quantification process and the possibility of the identification and chemical characterization of the analyte from the study of its vibrational features. The indirect detection correlates the SERS spectrum changes of a metabolite, reaction product, or reporter molecule (RM) with the concentration of the target analyte [28]. With this methodology, analytes with low or null Raman vibration modes can be detected, and multiplexed detection can be achieved [32]. The use of reporter molecules is the most common way to address indirect detection in biological samples. It consists of the functionalization of the substrates with one or several molecules (monoplexed or multiplexed detection, respectively) that present a change in their Raman cross-sections due to the interaction with the target analyte. RMs usually are small in size and present high Raman cross-sections. In addition, they are characterized by a narrow Raman spectrum or very few of their bands are superimposed with those from the matrix or the analyte spectra and are photochemically stable [33].

### 2.5. Determination of Viruses by SERS

The more sensitive and specific way to determinate viruses by SERS is using reporter molecules (indirect detection) combined with a sandwich immunoassay. The determination strategy by this route is basically integrated by: (1) a SERS tag, consisting of a Raman reporter molecule and the recognition element, which is a specific antibody (detection antibody) confined on SERS-active nanoparticles, and (2) a template, called the capture substrate (not necessarily a metal surface), which is functionalized with a linker antibody (capture antibody) to bind the antigen–SERS tag complex. The quantification is based on tracking the Raman signal from the reporter molecule before and after the SERS tag has interacted with the capture element [14]; this allows to overcome the intrinsic restrictions of using biological analytes in direct detection, such as large molecule sizes, low scattering cross-sections, weak affinities for the common noble metal SERS substrates, and low specificity [34,35].

## 3. Zoonotic Infections

Zoonotic diseases or zoonoses are those that can be transmitted from animals to humans [36]. The transmission occurs mainly by direct contact; by inhalation or ingestion of contaminated products; by touching an area or surface that has been infected by an animal; or by means of vectors, mainly hematophagous insects such as mosquitos, bugs, or ticks. Zoonoses are represented by a complex group of infectious diseases caused by a variety of microorganisms such as viruses, bacteria, fungi, protozoa, worms, and some insects of the arthropod type [37].

Currently, the World Health Organization (WHO) classifies zoonoses into three categories: (1) endemic: diseases that are present in many places and affect a high percentage of the population, both people and animals, (2) epidemic: zoonoses that are sporadic and localized in temporal and spatial distribution, and (3) emerging and reemerging: those that have appeared recently in a population as a consequence of a new interaction between people and wild animals or that have existed before but there is a significant increase in incidence or geographic range [38]. A study estimated that around one billion cases of disease and one million deaths due to zoonoses occur worldwide each year. In addition, 60% of the emerging infectious diseases reported worldwide are zoonoses, and in the last three decades, 30 new pathogens affecting humans have been detected, 75% of which have their origin in animals [39]. These data establish zoonoses as real health problems.

The increased transport of animals and people because of the current globalization, either for tourism or commerce, over long distances for short periods has led to the international spread of zoonotic infections. Many zoonoses are transboundary diseases; they spread across borders and impact trade, tourism, and consumer confidence, with devastating economic consequences [39]. In addition, the migration from urban areas to nonurbanized ones has caused the deforestation of jungles and tropical forests [40], mainly for agricultural development and poultry farming [41]; this has irremediably pushed wild animals closer to people, allowing zoonosis to occur.

**Figure 2:**
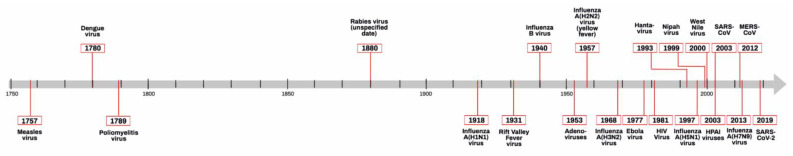
Timeline of zoonotic viruses from 1757 to the present day [46,47,48,49,50,51,52,53,54,55,56,57,58].

The severity and prognosis of zoonotic diseases vary depending on the type of disease transmitted. Many zoonoses are easily treatable, while others can produce acute, seriously acute, or chronic diseases, causing lifelong effects or even death, and unfortunately, there is no effective treatment or vaccine against several of these zoonoses. Two terms have been postulated to indicate how zoonotic diseases can be transmitted. Animal-to-human disease transmission is known as anthropozoonosis; for example, tuberculosis, yellow fever, polio, measles, influenza, and COVID-19. Zooanthroponosis is the opposite case, with examples such as giardiasis and infections by *Staphylococcus aureus* [42,43,44]. Viral anthropozoonosis has continuously been present in the history of diseases that represent a high risk for public health (Figure 2), since it is estimated that 75% of emerging infections fall into this category [45].

In general, viral zoonoses cause unknown infectious diseases in the new host, so there are no drugs or methods for their detection, since it is difficult to control their dissemination, and they become a potential pandemic risk. For this reason, several investigations have been carried out to elucidate the factors that allow viruses to acquire the ability to infect new hosts and spread efficiently [59].

Understanding how the successful transmission of a virus from an animal to a human or vice versa can result in a zoonosis or an endemic disease is complicated, due to the multifactorial nature involved in the process and because viruses are constantly evolving [60]. Most viruses possess RNA as genetic material, as is the case of influenza viruses and coronaviruses. The great genetic variety of this type of virus is mainly due to the absence of a viral reading mechanism of RNA polymerase; therefore, this type of virus can continuously generate errors when achieving replication cycles [61,62]. Recombination and mutation processes, known as viral diversity mechanisms, can be carried out during these replication cycles. Recombination and mutation are the keys for the interpretation of zoonotic diseases, since they favor the evolutionary processes for genetic diversity among wild species [63,64]. Recombination processes can commonly be produced by the frequent reassortment and rearrangement of genomes. Reassortment occurs when a coinfection is presented in a host, resulting in a new virus progeny with a new variety of genes as a consequence of the replacement of gene fragments. On the other hand, rearrangement refers to a novel combination of gene segments that results in a new variant of the virus. In regard to the mutation processes, these consist in the change of genetic sequences by substitution of single RNA blocks or nucleotide bases, giving rise to variants of the existing strains. Therefore, recombination and mutations can modify the antigenic variations in populations [62,65].

**Figure 3:**
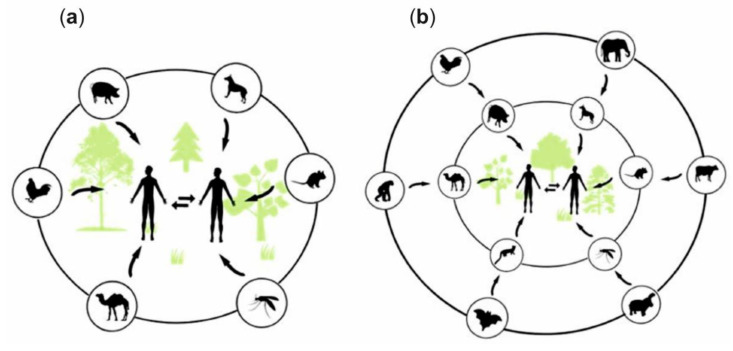
Scheme of the paths involved in the occurrence of an anthropozoonosis: (**a**) direct and (**b**) indirect infections.

The mechanisms of viral pathogenesis are triggered mainly by antigenic variations. These variations correspond to changes in viral proteins as a response to antibodies after infection. There are two types of variations: antigenic drift and antigenic shift. Antigenic drift (a random genetic mutation) results from slight alterations in the surface proteins of viruses. Under these variations, viruses can be transmitted to humans directly from infected animals, humans, or the environment (Figure 3a). Antigenic shift (a specific case of reassortment or rearrangement) is characterized by important modifications in the surface proteins of viral particles through the acquisition of new genes that encode new proteins or proteins that already exist [60,66,67]. Antigenic shift is less common, and it generally requires an intermediate host to achieve an adaptation to infect new hosts [68]. In this way, under antigenic shift variations, viruses can be transmitted from animals to humans indirectly (Figure 3b), provoking a higher number of infection cases, because the population does not have immunity against the latest virus [69,70].

## 4. Human Influenza Viruses

**Figure 4:**
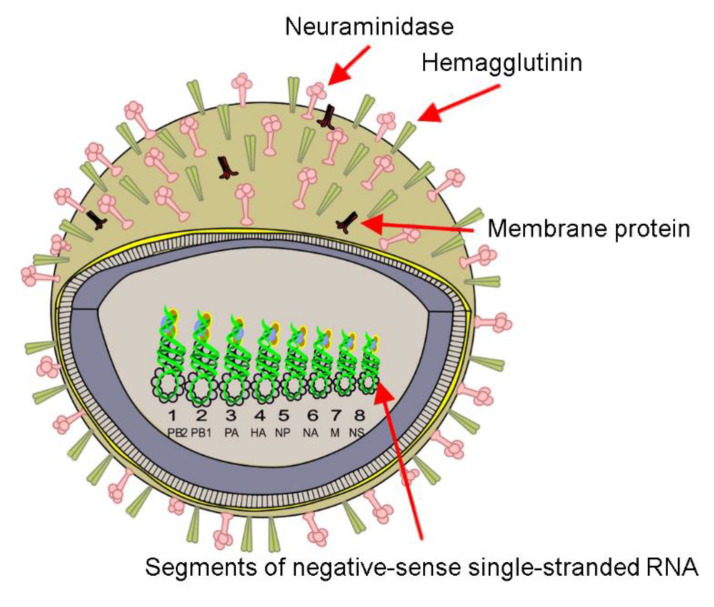
Scheme of the avian influenza virus.

Influenza viruses are among the main causes of respiratory tract infection in humans that, every year, provoke seasonal/endemic infections and, sometimes, unpredictable pandemics, resulting in about 650,000 deaths every year [71]. These viruses are associated with the *Orthomyxoviridae* family and are segmented negative-sense, single-stranded RNA viruses with an enveloped helical structure. Influenza virions present pleomorphism, which gives them the capacity to adopt spherical (the most common) and filamentous shapes, as well as variable sizes (150–400 nm) [72,73,74]. Four influenza viruses have been identified (A, B, C, and D). Influenza A, B, and C viruses affect humans and animals. Type D (a novel influenza C virus) is rare and mainly affects livestock, with no evidence that it may affect humans [75,76]. Influenza A, B, and C can cause zoonotic infections, but the A and B types are the current causes of the seasonal epidemics in humans [75,76,77]. Influenza A is cataloged as the more prone to generate epidemics and pandemics, having the deadliest flu pandemics in the human registry (Spanish flu or H1N1 influenza). As sketched in Figure 4, influenza A viruses possess eight segments of negative-sense single-stranded RNA viral and two surface glycoprotein spikes, hemagglutinin (HA) and neuraminidase (NA), embedded in the virus lipid bilayer (antigenic proteins). Influenza A viruses have 18 subtypes of hemagglutinin (H1–H18) and 11 subtypes of neuraminidase (N1–N11). Likewise, depending on the host from which the influenza A virus originates, it is classified as avian, swine, or other types [78].

### 4.1. Influenza A(H1N1) Virus (Spanish Flu)

The influenza A virus of the H1N1 subtype has been reported as a pandemic twice: in 1918 when the H1N1 strain emerged as a zooanthroponosis infection of swine origin [79]. It was known as “the Spanish flu”, although its origin was in New York [80]. With a registered duration of more than one year, the number of deaths caused by swine flu was estimated to be 100–500 million, with a Case Fatality Rate (CFR) of 2.5 percent [81]. Ninety-one years later, in April 2009, a new strain of the H1N1 swine flu emerged in the United States, according to the U.S. Centers for Disease Control and Prevention (CDC), although there is controversy if Mexico was the origin [82]. The new virus was labeled as A(H1N1) pdm09 or p(H1N1) and, in one year, caused about 575,400 deaths, with a CFR for symptomatic cases with medical attendance of about 0.05% [83]. As a relevant fact, the early data of influenza A(H1N1) reported that the infection rate was dependent on age, being high among young people between five and 24 years old [83]. In addition, advanced age patients or those with immune deficiency comorbidity were closely associated with severe outcomes and death [84]. In 2015, a mutant strain of A(H1N1) caused 774 deaths in India, and it was documented as a pandemic incidence [85].

#### SERS Detection of Influenza A(H1N1) Virus

Among the laboratory diagnostic techniques for H1N1 influenza detection, viral culture, rapid antigen test, direct immune fluorescence (DFA), and real-time PCR were found. Concerning alternative diagnostic methods, there is a wide variety of biosensors using antigens, enzymes, proteins, and DNA as the biological elements and thermal, piezoelectric, optical, or electrochemical transducer elements. For detailed information on these methods, the reader can be referred to the reviews by Dalal et al. [86] and Chauhan et al. [87].

Using SERS, the identification and quantification of different strains of influenza A(H1N1) have been carried out, as shown in Table 1 and Table 2. As observed in Table 1, identification studies have been attained in direct forms on solid substrates without using linker molecules: the analyte is set into contact with the SERS substrate through the entrapment of the target into the interstices formed among the metallic nanoparticles. In this way, the identification strategy is based on the obtaining of a Raman shift pattern coming from the particular viral lipid envelope segment of the strain; this is due to the fact that a complete characterization by SERS is not possible because of the pleomorphism and the large size of the A(H1N1) virus (150–400 nm) [74]; this restricts the obtaining of a maximum increase of the Raman signal through the electromagnetic mechanism.

**Figure 5:**
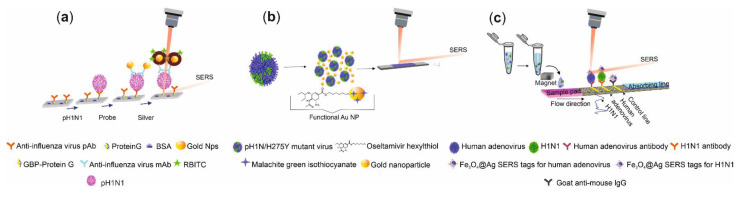
Operating procedure scheme of: (**a**) the immunoassay with protein G. Adapted with permission from ref. [93]. Copyright 2016 the Royal Society of Chemistry. (**b**) Functional nanoparticles system. Reproduced with permission from ref. [94]. Copyright 2019 American Chemical Society. (**c**) SERS-based lateral flow immunoassay strip. Reproduced with permission from ref. [95]. Copyright 2019 American Chemical Society.

The experimental details of SERS studies for the quantification of A(H1N1) influenza are summarized in Table 2. Moon et al. [93] developed a sensitive immunoassay that included the use of protein G to bind the antibodies on the capture substrate and on the gold nanoparticles (AuNps) to form antibody probes. The use of protein G allowed the specific immobilization of the antibodies, keeping its optimal conformation for an efficient interaction with the virus. The configuration included the conjugation of the antibody probes with the capture substrate that contained the analyte. Then, a silver cover was formed with the aid of an Ag-enhancer solution. Finally, the reporter molecules were immobilized on the silver cover (Figure 5a). For the selectivity analysis, they did experiments separately with the influenza viruses H3N2, H5N2, and influenza B (IBV), and the intensity of the signal located at 1643 cm^−1^ was at least 3.5 times higher for p(H1N1) than for the other viruses, thus confirming that the method has good selectivity.

G. Eom et al. [94] reported a dual detection by SERS and by Naked−Eye of oseltamivir−resistant virus −p(H1N1)/H275Y mutant− using functional gold nanoparticles. In this study, AuNps of 12 nm in diameter were functionalized with malachite green isothiocyanate (MGITC) as the reporter molecule, and oseltamivir hexylthiol (OHT) was employed to specifically link the p(H1N1)/H275Y mutant virus. The aggregation of AuNps driven by the presence of the p(H1N1)/H275Y mutant virus provided a strong SERS signal of MGITC, allowing the virus quantification (Figure 5b). It can be highlighted that the detection of p(H1N1)/H275Y in a sample containing 1% of this mutant virus in the presence of 99% of the wild virus demonstrated the high selectivity of the method. Likewise, C. Wang et al. [95] developed a SERS-based lateral flow immunoassay (LFIA) strip for the dual detection of influenza A(H1N1) and human adenovirus (HAdV) using magnetic SERS tags under the operating procedure shown in Figure 5c. A drop of a buffer solution that contained the desired magnetic SERS tag–virus complexes, formed in a previous incubation process, was set on the LFIA strip with two test lines. For positive samples, the sandwich immunoreactions occur in the respective test lines. A line for quality control was added to the strip to bind those SERS tags without the virus. The viruses were quantitatively detected through the main Raman peak at 1332 cm^−1^ from the Raman reporter on the SERS tags. Additionally, qualitative virus identification was possible by watching the color changes of individual test lines observed at a high viral tilter (about 105 plaque-forming units (PFU)/mL). The experiments made in either the influenza B (FluB), respiratory syncytial virus (RSV), or parainfluenza (PIV) samples did not show a significant response. On the contrary, tests carried out with a mixture of H1N1 and HAdV showed a significant signal for H1N1, and poor signal for the HAdV, in test line 1 and a significant signal for HAdV, and poor signal for H1N1, in test line 2, indicating a good selectivity of the system. The device was tested in real samples such as human whole blood and sputum without any sample pretreatment, envisaging its use in point-of-care testing.

### 4.2. Influenza A(H7N9) Virus

**Figure 6:**
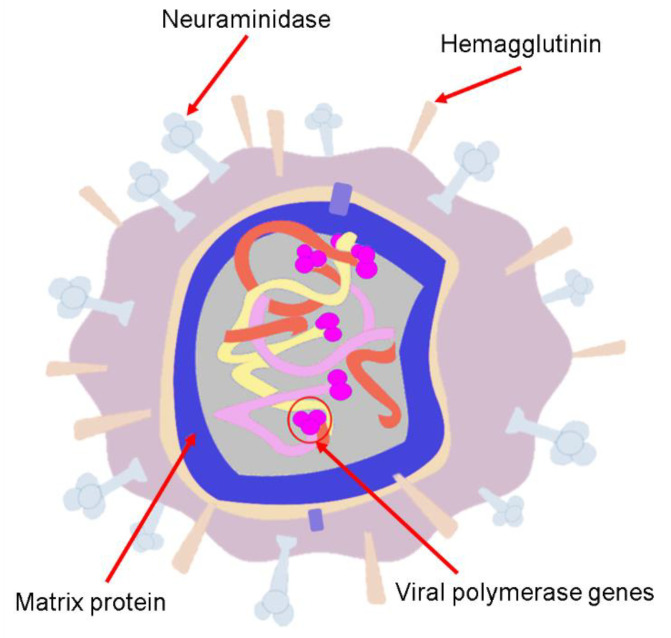
Scheme of the avian influenza H7N9.

In 2013, a new subtype of the avian influenza H7, known as A(H7N9) (Figure 6), was identified for the first time in humans in the provinces of Jiangsu, Zhejiang, Shanghai, and Anhui, China [96,97,98]. The H7N9 virus is considered of low pathogenicity [99], causing symptoms such as fever, cough, dyspnea, sore throat, encephalitis, and chest pain; however, the infection can *develop rapidly* and *attack the lower* respiratory tract, resulting even in mortality [100,101]. The severity of infection and death increased in pregnant women; adults *aged 65 or more*, and people with chronic and *underlying* health *conditions* (hypertension, diabetes, coronary heart disease, and immunosuppression) [102]. In the *first year* of the *H7N9 emergence,* 132 infected people *were accounted for* and 37 died in China. [103,104]. There have been seven epidemics reported of the H7N9 virus; the first four waves were of low pathogenicity (LPAI), and the subsequent three waves were considered highly pathogenic (HPAI) [105]. The change from LPAI to HPAI was due to the genetic shift in the surface of the hemagglutinin protein (HA). Some studies have found that most H7N9 viruses on the surface of the HA protein have dual receptor binding and the avian-like (α2,3-linked) and human-like (α2,6-linked) sialic acids [106], due to mammalian adaptive mutations, such as G186V and Q226L/I (number H3) [107,108]. The new H7N9 viruses from the fifth wave not only had the mammalian adaptation residues of the previous waves but also acquired additional virulence markers, such as PB2–A588V, and improved their pathogenicity [109]. In addition, the H7N9 HPAI viruses acquired four amino acid insertions (KRTA) and substitution in G320R (H7 numbering). These are also signs of adaptation to mammalian hosts by the acquisition of fusion capacity at a lower pH threshold than the other H7N9 viruses in HA, causing increased mortality. Therefore, HPAI H7N9 viruses experience greater virulence [105].

#### SERS Detection of A(H7N9)

**Figure 7:**
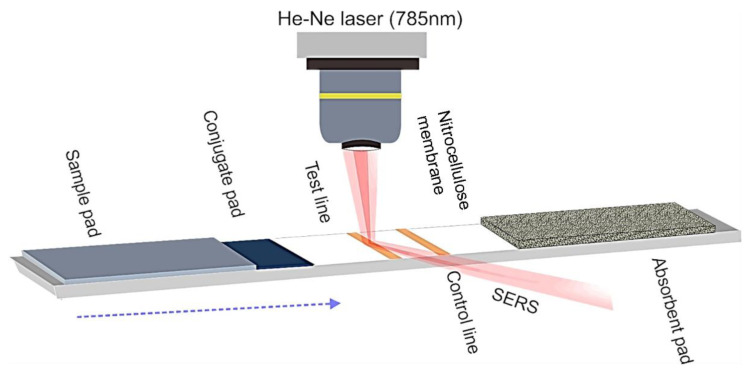
Surface-enhanced Raman scattering-based lateral flow immunoassay strips (SERS-LFIAS). Adapted with permission from ref. [106]. Copyright 2019 Elsevier Science S.A.

The diagnosis of this virus can be made through Rapid Influenza Diagnostic Tests (RIDT); however, commercially available kits cannot differentiate the subtypes and are less sensitive than PCR or real-time PCR, which are the routine methods for the identification of H7N9 influenza A(H7N9) [97]. However, despite real-time PCR being a sensitive and fast method for monitoring the H7N9 virus, working with high levels of amplification can generate false positives [106]. Due to these deficiencies, alternative techniques have been proposed for the detection of this disease, such as fluorescence [107], total internal reflection dispersion [108], electrochemistry [109], and enzyme-induced metallization [110]. Regarding the use of SERS, Xiao et al. [106] proposed the detection of inactivated H7N9 viruses using surface-enhanced Raman scattering-based lateral flow immunoassay strips (SERS-LFIAS), as shown in Figure 7. The SERS tags consisted of AuAg4_ATP@Ag core shell nanoparticles modified with the anti-H7N9 monoclonal antibody (H7N9-mAb). 4-aminothiophenol (4-ATP) was used as the reporter molecule and remained immobilized between the two silver layers covering the gold core, while the H7N9-mAb was immobilized in the outermost layer of silver. The test line and the control line of the LFIA strips were modified with H7N9-mAb and goat anti-mouse immunoglobulin G (IgG) antibody, respectively. When the H7N9 virus was present in the sample, H7N9-mAb-AuAg4_ATP@AgNps immunocomplexes were formed in the conjugation zone and migrated by capillary action into the test line, where they were captured. The Raman signal was recorded from the accumulation of the antibody–virus–antibody system. When the H7N9 virus was not present in the sample, excess mAb–AuAg4_ATP@AgNps complexes continued to migrate, and it was captured by the goat anti-mouse IgG antibodies present in the control line. Selectivity studies were carried out by measurements in different samples containing one of the following viruses: inactivated H5N1 (Re-6, Re-7, and Re-8); subtype H9 influenza virus; Newcastle disease virus-La Sota (NDV La Sota); or Infectious bursal disease virus (IBDV Gt). In these experiments, a 100% crossreaction rate was attained for H7N9, while less than 10% was shown for the other viruses. These results indicate a high selectivity. The limit of detection (LOD) for H7N9 was 0.0018 hemagglutination *units* (HAU). The feasibility of the developed quantification strategy was corroborated using spiked real samples such as cloacal and throat swabs, as well as organs of poultry spiked with different concentrations of the H7N9 virus.

**Figure 8:**
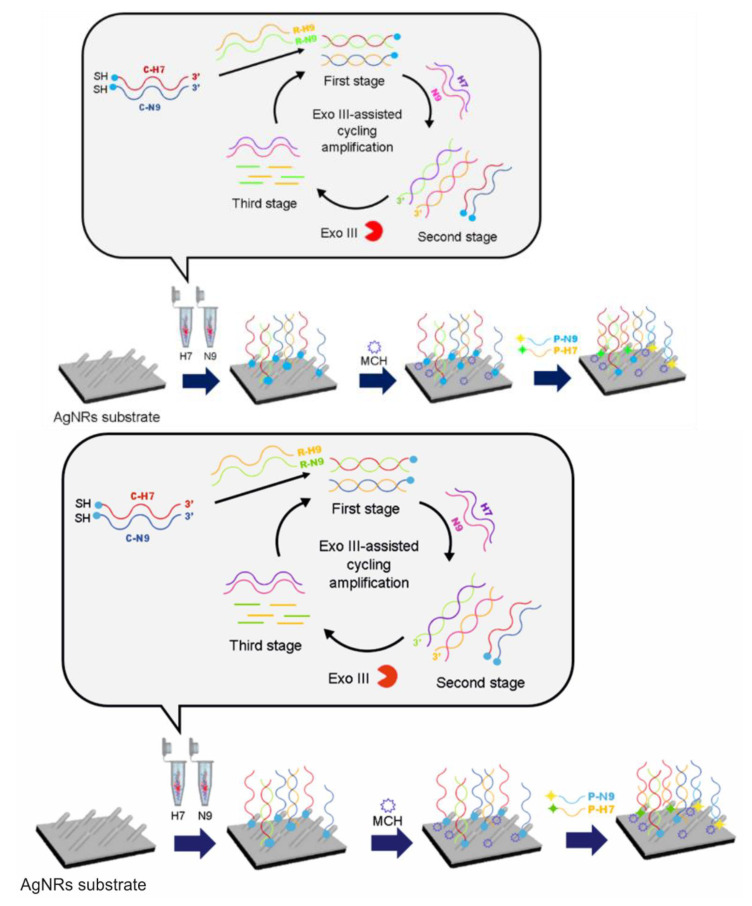
SERS quantification of the H7N9 virus, including the amplification process of the H7 and N9 gene fragments via reaction with exonuclease III. MCH: mercaptohexanol added to avoid nonspecific adsorption. Reproduced with permission from ref. [111]. Copyright 2019 Elsevier Science S.A.

On the other hand, Song et al. [111] proposed a sensitive SERS strategy for determining H7N9 based on a dual detection of the characteristic gene fragments of H7 and N9. The method contemplates the exonuclease III-assisted signal amplification of each gene, followed by their quantification by SERS (Figure 8). The amplification process was carried out independently for each gene by the specific design of a set of single-stranded DNAs, including capture, replace, and probe, to detect each gene fragment. The sensing mechanism was as follows: in the amplification process, the capture and the replace single-stranded (ss)DNAs were mixed together to form a double-stranded DNA. Then, the capture ssDNA was released by toehold exchange when the target (H7 or N9) was added. After that, the exonuclease III-assisted DNA cycling amplification was conducted in the presence of the exonuclease III enzyme, resulting in the release of the gene fragments of H7 or N9 to further trigger the toehold exchange with the capture/replace dsDNAs. Subsequently, the mixture was incubated on the SERS substrates consisting of small well-patterned silver nanorods (AgNRs) on a glass slide. The capture ssDNA released in the amplification process was immobilized onto the surface of AgNRs. Then, the immobilization of probe ssDNA was attained by its hybridization with probe ssDNA and incubation on the AgNRs substrate. The Raman signal coming from the H7 and N9 probes was obtained simultaneously on the same SERS substrates using parallel wells, and in this way, the concentration of the target was obtained. The SERS peak intensities at 1360 cm^−1^ and 1503 cm^−1^ were used to sense H7 and N9, respectively. The quantification presented a limit of detection as low as 31 aM for H7 and 44 aM for N9 and a recovery in the range of 93.8–106.2% in spiked samples containing different concentrations of H7 and N9 gene fragments. The signal obtained for H7 was significantly higher than the possible interferents, such as single-base mismatched sequence H7 (SM-H7), double-base mismatched sequence H7 (TM-H7), and noncomplementary sequence H7 (NC-H7); almost the same difference was obtained when comparing the signal for N9 with those for SM-N9, TM-N9, and NC-N9. In conclusion, this strategy presents a specific detection of H7 and N9 gene fragments.

### 4.3. Influenza A(H3N2) Virus

The introduction of the influenza A virus of H3N2 subtype into the human population occurred possibly in Hong Kong, China in July 1968. It was known as Hong Kong flu, and in its early stage, it was considered a mild disease, with symptoms of general malaise, fever, cough, headache, coryza, and sore throat, without a severe mortality record. The virus gradually became a global pandemic: its spread in Asia, Oceania, and the Middle East was confirmed in August. In September 1968, the virus was reported in the US, where it caused the most extensive mortality, with about 34,000 people out of one million accounted globally. In Europe, the virus was reported until the winter of 1969–1970. Complications in adult patients and those with comorbid conditions were mainly of the pulmonary kind, including localized primary viral or secondary bacterial pneumonia or diffuse bilateral pneumonia [112,113]. The virus was the product of a genetic reassortment between avian A(H3N2) and human A(H2N2) influenza viruses. Since 1968, the H3N2 influenza virus has been in constant evolution, mainly driven by antigenic drifts, modifying the surface antigens hemagglutinin and neuraminidase and, thus, preventing antibody binding; this has made recognition of the virus difficult. A new antigenic variation of the influenza A(H3N2) viruses can arise with a mean of two years, and it is this adaptation that allows the virus to predominantly be the cause of the flu today and the primary cause of seasonal influenza morbidity and mortality [114].

#### SERS Detection of A(H3N2)

**Figure 9:**
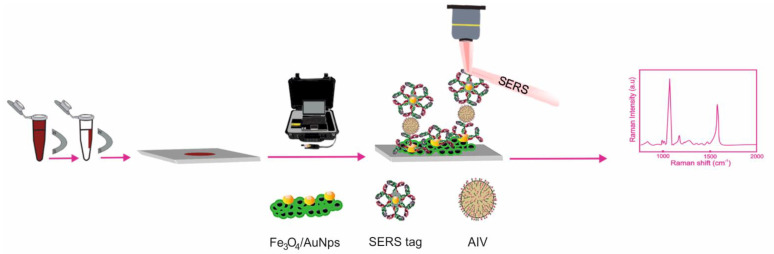
Schematic illustration of the SERS-based magnetic immunoassay for H3N2 virus detection. AIV: Type A influenza virus. Adapted with permission from ref. [119]. Copyright 2017 Elsevier Science S.A.

As in the rest of the influenza viruses, a PCR assay is the first-choice laboratory test for the detection of the A(H3N2) virus [115]. The alternative methods include electrochemistry [116], colorimetry [117], surface plasmon resonance (SPR) imaging [118], and surface-enhanced Raman spectroscopy. Sun et al. [119] reported the detection of inactivated A(H3N2) using a magnetic SERS immunosensor under a sandwich assembly (Figure 9). The SERS tags consisted of AuNps@4-MBA@Influenza A IgG. 4-mercaptobenzoic acid (4-MBA) served as both the Raman reporter molecule and the coupling agent to covalently bind influenza A IgG to the surface of the metallic particles through amide linkage. Gold-coated magnetic nanoparticles (Fe_3_O_4_/AuNps) modified with the antibody (A IgG) were used as the capture substrate. The sandwich immunoassay protocol consisted of mixing the SERS tags (AuNps@4-MBA@Influenza A IgG) and the supporting substrates (Fe_3_O_4_/AuNps @ Influenza A IgG) in a solution in the presence of different tilters of the H3N2 virus. Before incubation, the Fe_3_O_4_/AuNps as a complex with the analyte and the SERS tags were collected. The SERS measurements of the complex were deposited on aluminum foil and were conducted in a dry form. The quantification of the virus was carried out by using the band at 1583 cm^−1^, attributed to the υ8a aromatic ring vibration, as the marker band, since it increased with the increasing H3N2 concentration. The magnetic immunosensor detected H3N2 down to a LOD = 10^2^ TCID_50_/mL (TCID: median tissue culture infectious dose) with a good linear relationship (10^2^–5 × 10^3^ TCID_50_/mL). The specificity was tested by comparing the results of the virus with that of an inactivated H1N1 that was three times more concentrated, and it was found that the signal for H3N2 is approximately six times higher, which leads to inferring a good specificity.

**Figure 10:**
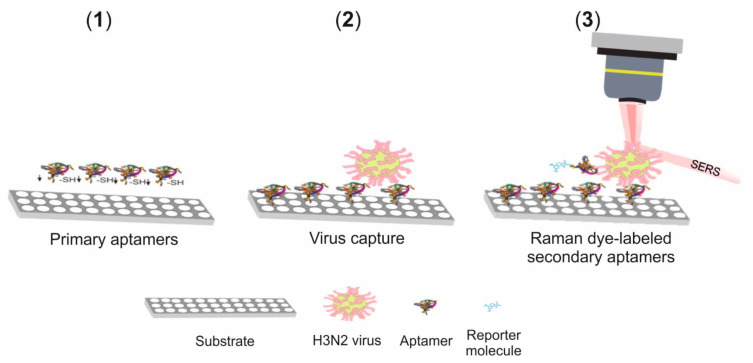
Scheme of the sandwich-like aptasensor for influenza virus detection. Steps: (**1**) immobilization of the aptamer onto Ag nanoparticles, (**2**) capture of the virus on the SERS substrate, and (**3**) interaction of the reporter molecule–aptamer complex with the virus. Adapted from ref [120].

Kukushkin et al. [120] developed a SERS-based aptasensor for the quantification of different strains of the influenza virus, including the H3N2 strain. They used the DNA aptamer RHA0385, which can recognize influenza hemagglutinins with highly variable sequences. In this way, the quantification proposal was not delimited to a particular strain of the influenza virus. Silver granules (20 nm size) were deposited in two circular regions (experimental and the control zones) on a silicon plate covered with a layer of SiO_2_. In the experimental zone, samples of allantoic fluid containing the influenza virus were tested, while allantoic fluid without the virus was used in the control zone. The control zone was used to confirm that the binding of some biomolecules of the sample does not represent a significant contribution to the virus quantification. The aptamer was immobilized via a thiol group on the surface of the silver granules in both zones. After that, the corresponding samples to be analyzed were put in contact with the surface of the experimental and control zones, followed by a staining with Cy3 dye, labeled RHA0385. The analytical signal in the experimental zone originated from the interaction of the reporter molecule (Cy3 dye) with the virus on the SERS substrate (Figure 10). The analytical curve was constructed from the intensity variation of the band at 1587 cm^−1^. The LOD was as low as 1 × 10^−4^ hemagglutination units per probe. It can be mentioned that the aptamer-based sensor is not specific for H3N2, since it shows a response to various influenza A viral strains, including the H1, H3, and H5 hemagglutinin subtypes.

### 4.4. Influenza A(H5N1)Virus

Among the human infections by influenza viruses derived from domestic and wild birds, H5N1 (designated as the A/Goose/Guangdong/1/96 virus) is a highly pathogenic avian influenza (HPAI) [121]. The clinical manifestations of the H5N1 influenza virus can be accompanied by respiratory (fever, cough, shortness of breath, and radiological evidence of pneumonia) and nonrespiratory symptoms (diarrhea, vomiting, and abdominal pain) [122]. This virus was identified in 1996 in domestic bird populations of poultry farms in Southeast Asia, where it was considered endemic. However, in 1997, it was detected for the first time in humans in the Guangdong Province of China [123,124]; this is considered the first recorded case of a purely avian virus that caused severe human illness and death, with six deaths out of 18 cases [125,126]. Sporadic outbreaks were recorded subsequently in 2003, 2005, and 2006 in many countries around the world [127,128,129,130]. In all outbreaks, the human infections caused by H5N1 were restricted to people associated with the direct handling of infected poultry, the slaughter or preparation of diseased poultry for consumption, the consumption of raw poultry products such as blood, or those in close contact with live poultry. The reported route of entry of the virus was through the respiratory or gastrointestinal tracts. In humans, the possibility of intestinal infection was supported by reports of H5N1-infected patients presenting diarrhea as the only initial symptom and patients reporting exposure to poultry [131,132,133]. Therefore, one effective strategy to contain the pandemic was the slaughter of all poultry in farms and markets.

The H5N1 avian influenza virus has a pathological origin that is likely to be multifactorial, involving increased competition for viral replication, viral diffusion, and differential responses to gene expression in infected host cells [133]. The evolution rate and positive selection of H5N1 viruses have increased due to vaccine-induced immune pressure in poultry or the subsequent adaptation after transmission to new hosts (e.g., bird-to-human transmission) [134]. Furthermore, a significant antigenic drift of avian H5 has been found in the vicinity of HA with key mutations in the antigenic A and B sites corresponding to H3 [135]. In the human influenza virus, the NS1 gene or its product contributes to virulence by allowing the virus to prevent the activation of the interferon response in the host [136,137]. On the other hand, currently, H5N1 viruses that cause lower respiratory tract infections are consistent with the presence of avian-type SA α-2,3 Gal receptors to which avian viruses can bind in human bronchiolar and alveolar cells [138,139].

#### SERS Detection of A(H5N1)

The rapidity of detecting the influenza A virus during the acute phase of infection is crucial to provide an adequate clinical treatment [140]. Given the nonspecific nature of the disease, laboratory confirmation of the H5N1 influenza virus is essential. However, laboratory confirmation of a diagnosis for H5N1 disease is often difficult; it requires a high index of suspicion and the most sensitive detection methods available (e.g., reverse transcriptase PCR) and may need multiple samples [141,142].

**Figure 11:**
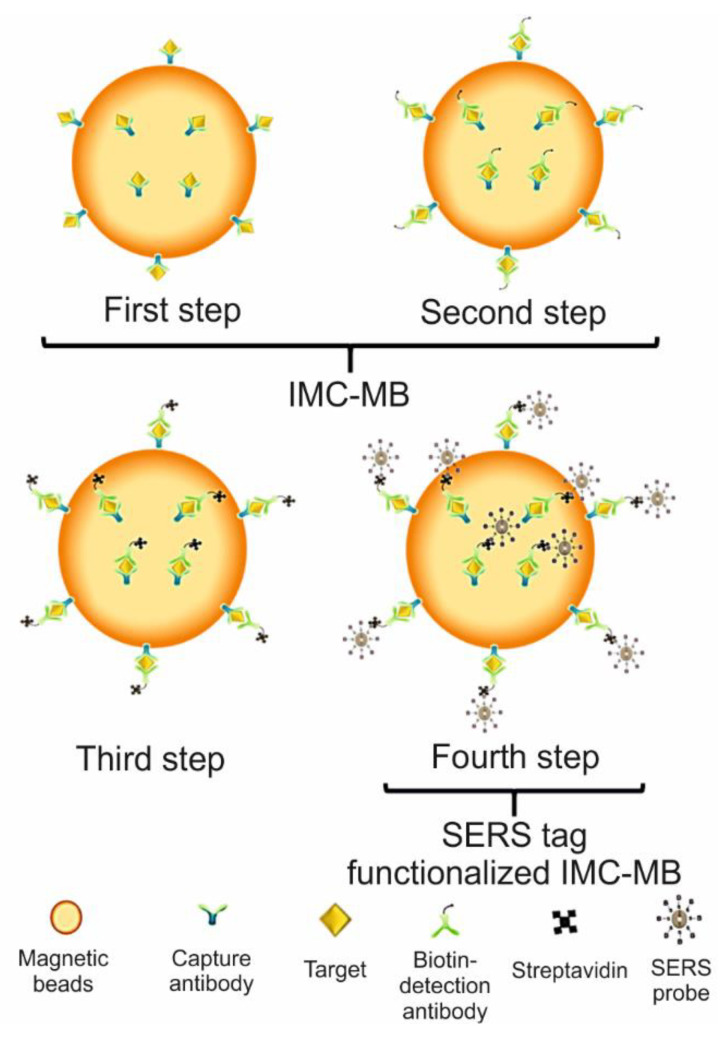
Representation of the steps to obtain the SERS tag-functionalized immunocomplex on which the SERS signal was recorded for the detection of the H5N1 virus. IMC-MB: immunocomplex on magnetic beads.

Regarding SERS studies, Wang et al. [143] reported the quantification of the H5N1 viruses on a digital microfluidic (DMF) platform, claiming that their study was the first report integrating a SERS-based immunoassay with DMF. The use of the DMF platform allowed the development of a lab-on-a-chip procedure and, consequently, of an automated procedure with certain benefits, such as the use of nanoliter sample volumes and less handling of biohazard samples. The SERS measurements were carried out under an immunoassay strategy, contributing to the sensitive and specific detection of the virus. The SERS immunoassay system consisted of the typical use of a SERS tag and of a capturing substrate. The SERS tag was composed of silver–gold core shell nanoparticles embedded with the reporter molecule, 4-mercaptobenzoic acid (4-MBA), and functionalized with the biotin antibody. As solid support, magnetic beads coated with H5N1 capture antibody (MB) were used. The DMF chip consisted of a confined droplet-based platform made of two parallel glass plates. The droplets were manipulated by applying sine wave potentials between the top and bottom plates. The bottom plate consisted of an electrode array of 30 active dispensing electrodes (1.7 μL of the volume capacity) and six reservoir electrodes (7 μL of the volume capacity), with interelectrode gaps of 20 mm. The top plate served as a ground electrode, and fluorocarbon oil (FC 40) was used as the oil phase to control and stabilize the drops. During measurements, five of the six reservoir electrodes were each preloaded with one of the following solutions: sample solution, washing buffer (Tris-buffered saline), biotinylated detection antibody (biotin-DA), streptavidin (SA), and biotinylated SERS tag (SERS probe); the sixth reservoir electrode was left for the waste solution. The analytical process consisted of loading a droplet of the solid support into the chip and mixing it, in four consecutive steps, with a drop of each substance from the reservoir. Each step was accompanied by an incubation process of five minutes, followed by the isolation of the beads (by their attraction to a magnet positioned underneath the chip) and by rinsing of the species formed with the MB. The unreacted reagents of each step were moved to the waste reservoir. The immunocomplex on the magnetic beads (IMC-MB) was prepared after mixing the MB with the sample (first step) and with the detection antibody (second step). The SERS tag-functionalized immunocomplex, on which the SERS signal is recorded, was produced after the successive interaction of the IMC-MB with streptavidin (third step) and SERS tag (fourth step), respectively (Figure 11). The DMF-SERS method showed good performance when evaluating the H5N1 antigen concentration in the serum with a LOD of 74 pg/mL, which is lower than that from the standard Enzyme-Linked Immunosorbent Assay (ELISA) method (399 pg/mL). Finally, the method showed good selectivity in the presence of the prostate-specific antigen (PSA), C-reactive protein (CRP), hepatitis B surface antigen (HBsAg), and cardiac troponin T (cTnT).

## 5. Coronaviruses

**Figure 12:**
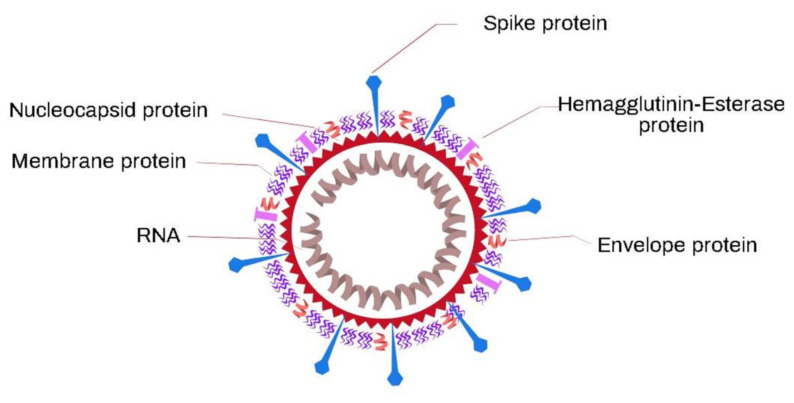
Scheme of the coronavirus structure.

Several animal species, such as birds and mammals, are the natural hosts of coronaviruses (CoVs). However, this type of virus is also capable of infecting humans, in which case, they receive the name of human coronaviruses (HCoVs). Coronaviruses are pleomorphic in structure and have one of the largest single-stranded RNA genomes of about 26–32 kilobase pairs and a unique replication strategy [68,144]. The structural characteristic that coronaviruses present is the spiked glycoproteins on the surfaces of the virions; these proteins make the virus seem like a solar corona, hence the name. These CoVs are known to be made up of a nucleocapsid and a positive single-stranded RNA (+ssRNA) as the genetic materials [145,146,147] that are surrounded by a lipid bilayer consisting of various proteins associated with the attachment to the cell that is invaded. The integral membrane proteins include spike (S), hemagglutinin esterase (HE), envelope (E), membrane (M), and nucleocapsid (N) (Figure 12). Among these integral proteins, the S glycoprotein spike is the one that plays an important role in the initial stage of CoV infection. This glycoprotein is made up of two structural subunits: the S1 and S2 regions. The S1 region forms the distal part of the spike and interacts with the host receptor—in humans, the angiotensin-converting enzyme-2 (ACE-2)—through the receptor-binding domain (RBD); from this contact, the infectious process begins. The S2 region anchors the spikes in the viral envelope and mediates the fusion of the virion and cell membranes. Consequently, the spike protein is the most divergent among all the coronavirus proteins [148].

On the other hand, when replication in the cytoplasm of the cells occurs, these viruses attack by nests or sets of RNA chains; for this reason, they are classified as nidoviruses. Coronaviruses belong to the *Coronaviridae* family and the *Coronavirinae* subfamily. The *Coronavirinae* subfamily consists of four genera based on their phylogeny and genomic structures: alpha-CoV, beta-CoV, gamma-CoV, and delta-CoV [60]. The major natural reservoir of the alpha-CoV and beta-CoV is mammals (mainly bats) [60,149,150]. The gamma-CoV and delta-CoV infect bird species, and, under certain conditions, some of them can also infect mammals such as beluga whales and pigs, causing them intestinal infections [148,150].

HCoVs were first detected in the US in 1966 from the nasal secretions of a patient with rhinitis [151,152]. After that, seven other strains of HCoVs have been so far reported, including human coronavirus 229E (HCoV-229E, 1966), human coronavirus OC43 (HCoV-OC43, 1966), severe acute respiratory syndrome coronavirus (SARS-CoV, 2003), human coronavirus NL63 (HCoV-NL63, 2004), human coronavirus HKU1 (HCoV-HKU1, 2005), Middle East respiratory syndrome coronavirus (MERS-CoV, 2012), and the ongoing severe acute respiratory syndrome coronavirus 2 (SARS-CoV-2, 2019) [153,154,155]. HCoV-229E, HCoV-Nl63, HCoV-HKU1, and HCoV-OC43 typically induce mild or moderate damage to the upper respiratory tract, without epidemic risks [150,155,156,157,158,159]. SARS-CoV and MERS-CoV were considered new types of coronaviruses that presented major pathogenicity compared to their predecessors and resulted in severe respiratory syndrome. In 2003, the so-called SARS-CoV was the agent responsible for causing an epidemic outbreak in China and the African and Southeast Asian countries [60,160]. From phylogenic studies, it is accepted that SARS-CoV originated in bats and that its subsequent transmission to humans happened by intermediate hosts such as civets, which share the same natural habitat with bats [161,162,163]. In 2012, the MERS-CoV virus caused a Middle East epidemic (the United Arab Emirates, the Kingdom of Saudi Arabia, Oman, and Jordan) and of two Asian countries (Thailand and South Korea). Currently, zooanthroponosis is the accepted origin of the virus. Phylogenic studies on the strains related to MERS-CoV have shown the bat as the origin and the camel as the intermediate host [164,165,166,167]. Finally, the SARS-CoV-2 was detected for the first time in December 2019 in Wuhan, China and produced a new severe respiratory syndrome that propagated rapidly across the globe [62,160]. The viral pathogenesis of SARS-CoV-2 is so far unknown; however, recent phylogenetic analyses have revealed that SARS-CoV-2 is genetically similar to the SARS-CoV and MERS-CoV viruses, with 80% homology. On the other hand, the complete genome sequencing in samples of infected patients showed a lineage that is very close to the bat coronavirus [158,168,169]. In addition, since this virus could be close to an 80% recombinant from some bat strains, the rest could come from other species, maybe the intermediate host [158].

### 5.1. SARS-CoV-2 Virus

**Figure 13:**
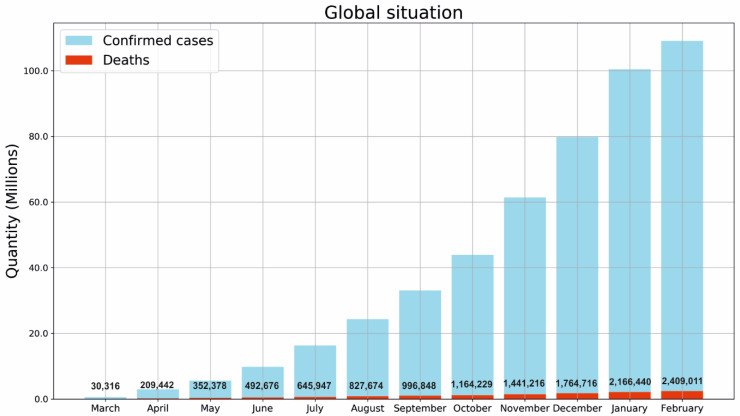
Cumulative confirmed cases and deaths related to the COVID-19 disease [173].

In December 2019, a novel coronavirus that has been named SARS-CoV-2 was detected in Wuhan, Hubei Province of China, presumably originating from a local market for seafood and exotic animal trade. SARS-CoV-2 is the seventh coronavirus known to infect humans and provokes the infectious disease named COVID-19 [158,170]. In March 2020, the World Health Organization (WHO) declared COVID-19 as a pandemic [146]. The number of confirmed cases and deaths reported globally is shown in Figure 13. The mortality rate of this disease has been estimated as 4.5% [171]. With the current estimate of the total world population (7700 million) [172], the COVID-19 pandemic represents a significant risk of spread.

The infected patients do not always express specific clinical symptomatology, which causes a significant difficulty in the correct diagnosis. On the contrary, symptomatic people present diverse clinical signs ranging from moderate to severe; among these: fever, dry cough, diarrhea, vomiting, abdominal pain, dyspnea, myalgia, fatigue, headache, pharyngeal pain, rhinorrhea, and severe pneumonia that can lead to death [174,175]. Currently, the treatment of infected patients is only supportive, trying to reduce the discomfort associated with the disease and reduce the complications or sequels that could originate. The asymptomatic patients still can spread the virus, a situation that has favored the propagation of the disease [58,175].

On the other hand, it has been found that symptomatic patients who are third-age adults and possess compromised immune systems or comorbidities such as diabetes, hypertension, and obesity are prone to suffer complications of shock and multiple organ failure, having, in some cases, a fatal outcome. Based on the currently confirmed cases, the most common age range of infected patients has been reported as between 30 and 79 years, and about 65% are men [175,176]. The basic reproduction number (R_0_) of the virus was estimated to be around 2.2, which means that each infected person can generate 2.2 new cases on average [177].

Since a vaccine could end the pandemic generated by this virus, this has been a priority for various research groups and the pharmaceutical industry. At the time of writing this review, several vaccine prototypes have been satisfactory in phase III clinical trials; thus, their application to the most susceptible population has been approved and has already started. The first approved vaccine was the one made by Pfizer-BioNTech [178], called BNT162b2, which is based on a messenger RNA (mRNA) in the same way as the second one, prepared by Moderna and named mRNA-1273 [179]. Both vaccines were authorized for emergency use. The third approved vaccine was developed by the University of Oxford and AztraZeneca, called ChAdOx1 nCoV-19 [180], and was developed under the platform of an adenovirus vector. In this race to obtain an effective vaccine, several investigations carried out by institutes such as Bharat Biotech, CanSinoBIO, the Gamaleya Research Institute, Sinopharm, Sinovac, Inovio Pharmaceuticals, and the Vector Institute [181] are in clinical phase III or about to be approved for application, either fully or for emergency use. These vaccines are being designed on various platforms such as live attenuated viruses, recombinant proteins, bacterial vectors, and DNA [181,182].

Undoubtedly, the implementation of various vaccines against SARS-CoV-2 will set a precedent due to the speed with which an effective vaccine was obtained and will help to significantly reduce the number of infected people. However, it is also necessary to improve diagnostic techniques to have a significant advance.

**Figure 14:**
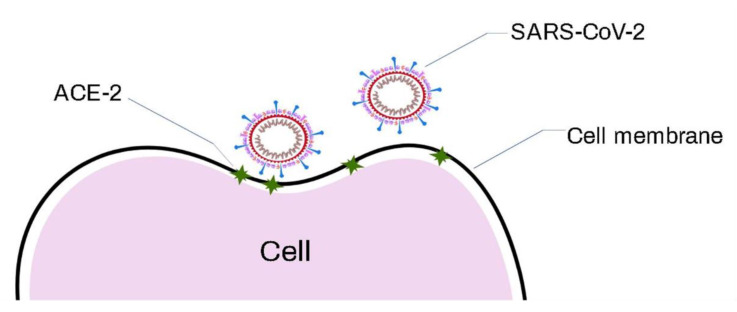
Representation of the severe acute respiratory syndrome coronavirus 2 (SARS-CoV-2) virus recognition by the ACE-2 enzyme, before its entry into the cell.

Studies about the genetic diversity (phylogenetic studies) and the possible origin of SARS-CoV-2 showed that the ancestors of SARS-CoV-2 are the coronaviruses, including SARS. Rehman et al. [60] reported that the complete SARS-CoV-2 genome sequence has approximately 80% nucleotide similarity to epidemic SARS viruses. The structural proteins remained conserved, except for the spike protein (protein S), which presented a high mutation rate in SARS-CoV-2 [60,183,184]. Likewise, they showed that approximately 81% of the amino acids of the SARS-CoV-2 protein S presented poorly conserved patterns with respect to other HCoVs [58,59,60]. The literature suggests that mutations in different genomic regions of SARS-CoV-2 have a specific influence on viral adaptation and production. Thus, through a natural evolution mechanism, the virus must have acquired mutations for satisfactory binding with the human ACE-2 receptor, as seen in Figure 14 [158,159,185].

Systematic analyses of the protein profile have shown a high amino acid (aa) substitution degree in the SARS-CoV-2 virus, indicating that viral proteins are heterogeneous. It has been found that receptor-binding domain (RBD) residues that interact with the enzyme ACE-2 and the crossreactive neutralizing antibody remain conserved in the various virus strains analyzed. On the other hand, according to the preliminary epidemiological data on SARS-CoV-2 infections, the frequency of aa mutations is higher in the sequences of the SARS-CoV-2 genome in Europe (43.07%), Asia (38.09%), and North America (29.64%), and the highest fatality rates were in temperate European countries, such as Italy, Spain, the Netherlands, France, England, and Belgium. Therefore, this genome analysis could be a promising tool for monitoring and tracking the associated genetic variants and their consequences [186].

In general, for coronaviruses, once the virus interacts with the receptor, it invades the cells until reaching the cytoplasm, where their replication begins; cellular compartments such as the endoplasmic reticulum (ER) and the intermediate compartment of the endoplasmic reticulum Golgi apparatus (ERGIC) have been observed to undergo various changes. These changes involve the contribution of the host cell membranes and organelles to viral replication. After the internalization and release of RNA in the cytoplasm, a set of proteins are synthesized and favor the formation of vesicles that become a viral platform that ensures the efficient replication and transcription of RNA. The new viral particles are assembled in the endoplasmic reticulum and the Golgi complex; then, the new full–length virions are delivered to the extracellular environment following a conventional secretion pathway [144,187,188].

Regarding the future behavior of COVID-19, Lavine et al. [189] carried out an analysis of the immunological and epidemiological data about endemic HCoVs and found that disease-reducing immunity is long-lived. Their model, incorporating these components of immunity, evaluated both the current severity of SARS-CoV-2 and the benign nature of HCoVs, suggesting that, once the endemic phase is reached and primary exposure is in childhood, SARS-CoV-2 could be less virulent, similar to a common cold.

#### SERS Detection of SARS-CoV-2 Virus

The analytical method currently used for the detection of the SARS-CoV-2 virus consists of a specific diagnosis made by using the real-time PCR of respiratory samples such as an oropharyngeal swab, nasopharyngeal swab, bronchial alveolar lavage, or tracheal aspirates. The samples are taken from a nasal exudate from both nostrils using a large swab introduced to the nasopharynx and subsequently rotated in a circular path at least four times for a total of 15 s [190].

**Figure 15:**
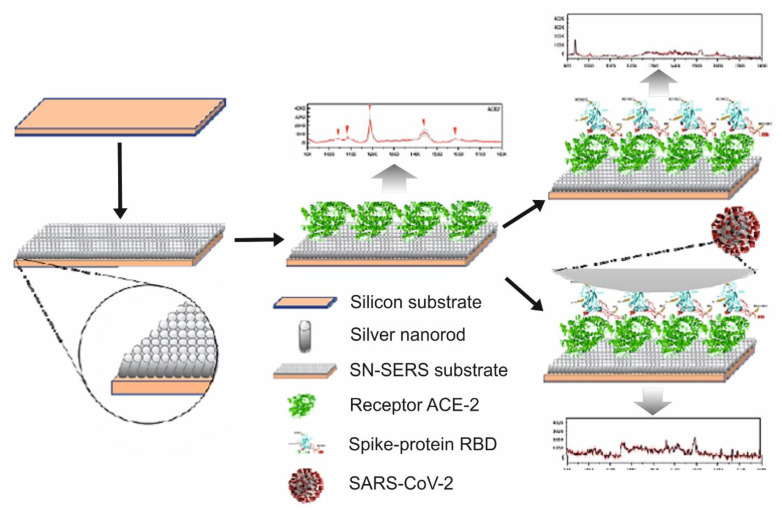
Scheme of the analytical strategy for the diagnosis of SARS-CoV-2 by SERS through the recognition of their spike protein receptor-binding domain (RBD) by the ACE-2 protein. The response of the analytical system in the presence of a free spike protein is also shown [194].

The technique uses two different amplification regions of the genes or primers of the E protein (envelope protein), NP (nucleoprotein), and the RNA-dependent RNA polymerase (RpRd) [191,192]. However, nucleic acid assays for COVID-19 present the limitation that they require high-quality viral RNA in high quantities (these amounts vary greatly between patients) and the high risk of viral RNA spoilage during collection, transport, and storage. On the other hand, despite the high sensitivity of the PCR technique, a negative nucleic acid test is insufficient to exclude SARS-CoV–2 infection in patients with high clinical suspicion. If a negative result in the nucleic acid test is observed once or twice, other alternatives must be considered for a correct diagnosis, such as an ELISA serological study for IgG and IgM antibodies designed by using the nucleoprotein of the bat coronavirus [187,188,189,190,191,192,193]. SERS is one of the novel alternative emerging techniques that can be used for the quantification of SARS-CoV-2 due to its proven performance in real samples of biological nature, including virus detection, as we have evidenced in this review. Zhang et al. [194] explored the diagnosis of SARS-CoV-2 using SERS combined with a multivariate statistical analysis. In this analytical system (Figure 15), the human cellular receptor ACE-2 was *physisorbed* on aligned silver-nanorod SERS arrays deposited on silicon wafers (ACE-2@SN-SERS substrates). The ACE-2 enzyme is recognized as a functional receptor for the spike glycoprotein of the human coronavirus SARS-CoV-2, specifically in its S1 subunit [185]. Thus, ACE-2 worked as the molecular recognition element but at the same time as the reporter molecule; this was because its Raman signal obtained *using* an *excitation wavelength* of *780 nm* was quenched when the recognition and binding of the RBD of the SARS-CoV-2 spike protein occurred. Using the ACE-2@SN-SERS assay, several real water samples from hospitals and pipe networks—taken before and after different processes of biological wastewater treatment—were on–site tested without any pretreatment. The presence or absence of the SARS-CoV-2 virus in the samples was previously corroborated by real-time PCR. In regard to the diagnostic of the SARS-CoV-2 spike protein in the water samples, it was based on induced SERS signal quenching in the presence of the virus, using two indicators to classify positive and negative samples. The first one, based on the behavior of the band at 1182 cm^−1^ assigned to amide II for C–N stretching and N–H bending of the ACE-2 enzyme, in the presence of the SARS-CoV-2 spike protein, undergoes a red shift to 1189 cm^−1^ as a result of a change in the ACE-2 structure when the binding occurs. The ratio of Raman intensity at 1182 cm^−1^ to that at 1189 cm^−1^ (1189/1182 ratio) was used as a biomarker. The second one, based on the construction of principal component analysis with linear discriminant analysis (PCA-LDA) score plots of the whole spectral alterations of the samples, used the first LDA score (LD1 score) between the positive and negative groups. From the results using both indicators, the diagnosis of the SARS-CoV-2 spike protein is comparable with that from real-time PCR, except for samples treated by a biological process, where PCR did not detect the virus, contrary to the SERS results of the 1182/1189 ratios and LD1 scores. This was explained by a higher stability of the SARS-CoV-2 spike protein against disinfection compared to RNA. With this technique, only positive or negative results were obtained; thus, an LOD value was not reported. On the other hand, the lack of a study of interference or selectivity against other ACE-2 targeting viruses, and the fact that the assay responds to free spike proteins or the viral envelope, which can lead to an overestimation of the presence of SARS-CoV-2, are some of the limitations of this methodology.

## 6. Conclusions and Perspectives

Due to the current pandemic situation and the constant threat of new infectious diseases of zoonotic origin caused by viruses, the need to develop new analysis techniques for their diagnosis has become relevant. The SERS strategies used for the identification of various subtypes of influenza and the new coronavirus SARS-CoV-2 were analyzed in this review. Through the direct method, the identification of several strains of the influenza A(H1N1) virus was addressed by the entrapment of the viral lipid envelope segment in the interstice between metallic nanoparticles without the need for linker molecules. However, this methodology lacks specificity, because different viruses or species of the same size can be trapped and detected; thus, pretreatment of the samples would be necessary. In addition, since viruses present pleomorphism, only those with adequate size are trapped within the nanoparticles, which represents a decrease in the sensibility of the detection. These facts represent a serious limitation for its practical use. Through the indirect method, the quantification of influenza has been achieved by using mainly the classic strategy of the sandwich immunoassay (SIA); SIA integrated with a digital microfluidic platform (DMF) has been employed for the detection of the influenza A H5N1 virus and lateral flow SIA for the dual detection of the influenza A(H1N1) virus and human adenovirus (HAdV). Other methodologies contemplate SERS and Exo III-assisted cycling amplification for influenza A H7N9 quantification and the design of a SERS-based aptasensor employing a DNA aptamer (RHA0385) for influenza A H3N2 quantification. Regarding the novel SARS-CoV-2 virus, its quantification was conducted using SERS combined with a multivariate statistical analysis through the detection of the receptor-binding domain of the spike protein. In this context, it can be inferred that the indirect SERS methods, especially those based on SIA, have good possibilities of reaching a position as a point-of-care technology for the diagnosis of viral infectious diseases, such as influenza and the coronavirus. Likewise, novel analytical strategies are perceived for the future, such as multiplex assays that incorporate the detection of RVsZO together with their specific biomarkers or secondary disease biomarkers resulting from the infection progress. The above-mentioned configurations could be used as a test of double confirmation or to screen the health conditions of the patient.

## Figures and Tables

**Table 1:** Representative studies of the direct identification of the A(H1N1) influenza virus by surface-enhanced Raman spectroscopy (SERS).

Name of Flu Virus	Virus Concentration (PFU/mL)	Excitation Laser (nm)	Type of Raman Measurement	SERSS ubstrate	Route of Identification	Ref.
A/WSN/33 (H1N1) ^†^	1 × 10^6^	633	Wet	Au/Ag multilayered nanorod arrays onto SCS *	Virus entrapment	[88]
A/WSN/33 (H1N1) ^†^	1 × 10^4^	633	Dry	Au substrates onto SCS *	Virus entrapment	[89]
A/Taiwan/N39/06 (H1N1)	1 × 10^6^	633	Wet	Au nanorods onto SCS *	Virus entrapment	[90]
A/WSN/33 (H1N1) ^†^	1 × 10^4^	633	Wet	Au nanorods onto SCS *	Virus entrapment	[91]
A/California/04/2009 (H1N1)	–	785	Dry	Aggregates of spherical AuNps on cover glass	Virus entrapment	[92]

^†^ Common laboratory strain. * Single-Crystal Silicon. AuNps: Gold nanoparticles. PFU: plaque-forming unit.

**Table 2:** Representative studies of the indirect quantification of A(H1N1) influenza virus by SERS.

Virus	LOD	Laser (nm)	Strategy	SERS Tag	Capture Substrate	RM	Tracking Band (cm^−1^)	Selectivity	Ref.
A/CA/07/2009 p(H1N1)	4.1 × 10^3^ TCID/mL	632	Immunoassay	AuNps−Ag−protein G−mAb−RBITC	pAb−Cys3−protein G−glass substrate	RBITC	1643	H3N2, H5N2, IBV	[93]
p(H1N1)/H275Y mutant	10 PFU	633	Functional nanoparticles	AuNp−OHT and MGITC	–	MGITC	1616	p(H1N1) *	[94]
A/FM/1/86 (H1N1)	50 PFU/mL	785	Immunoassay	Fe3O4–DTNB/@Ag –DTNB–antibody	pAb–LFIA strip of nitrocellulose membrane	DTNB	1332	HAdV, FluB, PIV, RSV	[95]

TCID: tissue culture infectious dose, RBITC: rhodamine B isothiocyanate, IBV: influenza B (Yamagata strain), OHT: oseltamivir hexylthiol, MGITC: malachite green isothiocyanate, DTNB: 5,5−dithiobis−(2−nitrobenzoic acid), p(H1N1) *: wild type, FluB: influenza B virus, PIV: parainfluenza virus, RSV: respiratory syncytial virus, HAdV: human adenovirus, and RM: reporter molecule.

## Data Availability

The data presented in this study are available on request from the corresponding authors.
